# Item Analysis of Otorhinolaryngology (ENT) Multiple-Choice Questions in an Internal Assessment Among Undergraduate Medical Students at a Rural Medical College in Maharashtra, India: An Analytical Cross-Sectional Study

**DOI:** 10.7759/cureus.105651

**Published:** 2026-03-22

**Authors:** Mohammad Ghodke, Shahrukh Ausekar, Purshottam Giri, Azhar Siddiqui

**Affiliations:** 1 Community Medicine, JIIU's Indian Institute of Medical Science and Research Medical College, Jalna, IND; 2 Otorhinolaryngology, JIIU's Indian Institute of Medical Science and Research, Jalna, IND; 3 Anatomy, JIIU's Indian Institute of Medical Science and Research Medical College, Jalna, IND

**Keywords:** analytical, cross-sectional, ent, ideal criteria, part completion test

## Abstract

Background: Multiple-choice questions (MCQs) are widely used for the assessment of knowledge in undergraduate internal assessment, but systematic post-test evaluation is required to maintain the quality of MCQ items. This study aimed to evaluate an otorhinolaryngology (ENT) internal assessment MCQ paper using difficulty index, discrimination index, and distractor efficiency to identify items for retention, revision, or discard to strengthen the departmental MCQ bank.

Methods: An analytical cross-sectional item analysis was conducted for the seventh-semester MBBS ENT Part Completion Test at JIIU's Indian Institute of Medical Sciences and Research Medical College, a rural medical college in Maharashtra, India. All students who appeared for the exam were included (n = 138). The test comprised 30 single best-answer MCQs (one key and three distractors). There was no negative marking. Difficulty index (p-value), discrimination index (DI, extreme-group method; upper and lower 27%), and distractor efficiency (DE; non-functional distractor defined as <5% selection) were calculated. Items were categorized as retain/revise/discard based on prespecified rules.

Results: The mean total score was 10.76 ± 5.36 (range 4-28). The mean difficulty index was 0.359 (range 0.072-0.833); 15/30 (50%) items were difficult (p < 0.30), 14/30 (46.7%) were of moderate difficulty (0.30-0.70), and 1/30 (3.3%) was easy. The mean discrimination index was 0.425; 16/30 (53.3%) items showed good discrimination (DI ≥ 0.40), 8/30 (26.7%) acceptable (0.20-0.39), and 6/30 (DI < 0.20) poor discrimination, including three items with negative discrimination (Q6, Q7, and Q25). The mean distractor efficiency was 90.0%; 23/30 (76.7%) items had DE = 100%, 5/30 (16.7%) had DE = 66.7%, and 2/30 (6.7%) had DE = 33.3%. Five items (5/30; 16.7%) met the predefined desired criteria (moderate difficulty, DI ≥ 0.40, DE = 100%). Overall, 10 items were retained, 17 recommended for revision, and three discarded.

Conclusion: The ENT internal assessment contained many items with good discrimination and strong distractor efficiency, but the difficulty index was skewed toward difficult items. Three items showed negative discrimination. Routine item analysis with structured revision can strengthen the departmental MCQ bank and support local assessment quality improvement.

## Introduction

Assessment of knowledge is an integral part of medical education. There are different methods of assessment as per the National Medical Commission (NMC) Competency-Based Medical Education (CBME) curriculum; multiple-choice questions (MCQs) are one such method. Single best-answer MCQs consist of a stem and two or more option choices from which examinees must choose the correct response (the key) and the remaining incorrect but plausible options (distractors) [1]. The MCQ format allows teachers to efficiently assess large numbers of candidates and to test a wide range of content [1]. If properly constructed, MCQs are able to test higher levels of cognitive reasoning and can accurately discriminate between high- and low-achieving students [1]. Evaluation of the quality of MCQs can be done by item analysis. Item analysis is the process of collecting, summarizing, and using information from students’ responses after conducting a test based on MCQs [2]. It analyses the performance of an individual MCQ item and the overall performance of the MCQ test [2]. MCQ writing has historically been viewed as a loosely organized set of guidelines often based on personal experience with limited empirical research [3]. Item analysis is not routinely implemented across departments in many settings, and published reports focusing on otorhinolaryngology internal assessments, particularly from rural medical colleges, are limited. Periodic item analysis across different batches will enable teachers to build a pool of high-quality items, strengthen the departmental MCQ bank, and obtain feedback for improving MCQ construction [4,5].

Accordingly, this study evaluated otorhinolaryngology MCQs used for the assessment of knowledge in MBBS Phase III Part II internal assessment (Part Completion Test) at a rural medical college in Maharashtra, India. It used standard item analysis parameters, i.e., difficulty index, discrimination index, and distractor efficiency, to generate evidence-based recommendations for the retention, revision, or discard of items for improving the departmental MCQ bank.

## Materials and methods

This analytical cross-sectional study used the post-test item analysis of an existing otorhinolaryngology Part Completion Test conducted for seventh-semester MBBS students at JIIU's Indian Institute of Medical Sciences and Research Medical College, a rural medical college in Maharashtra, India. All students who appeared for the test were included (n = 138). No student-level exclusions were applied. For analysis, responses were anonymized. Analysis was performed at the item level using anonymized responses, and no individual-level reporting was done. The institutional ethics committee (IEC) reviewed the proposal and granted exemption as the study used anonymized, routinely collected internal assessment data with no identifiers and no intervention. The written question paper comprised 30 single best-answer MCQs (see Appendix). Each item had one key (the correct response) and three distractors (incorrect responses). Students marked one option per item. Each correct response carried 1 mark. There was no negative marking. The time allotted was 30 minutes. To reduce copying, the same items were administered in four versions with a different item sequence. The difficulty index (p-value) was defined as the proportion of students answering an item correctly. Items were classified as easy (>0.70), moderate (0.30-0.70), or difficult (<0.30) [6]. The difficulty index is calculated as Diff index p = C/Ntotal, where C = number of students who answered the item correctly and Ntotal = total number of students who attempted the test. The discrimination index (DI) was calculated using the extreme-group method. After ranking total scores, the upper 27% (high achievers) and lower 27% (low achievers) were selected (rounded to 37 in each group) [7,8]. DI computed as DI = (H - L)/n, where H = number of students answering correctly in the high-achiever group, L = number of students answering correctly in the low-achiever group, and n = number of students in each extreme group. The DI ranges from -1 to +1. Items were classified as poor (<0.20), acceptable (0.20-0.39), and good (≥0.40).

Distractor analysis was performed by examining the frequency of selection of each distractor (excluding the key). A distractor selected by <5% of students was considered a non-functional distractor [1]. Distractor efficiency was calculated as DE = (number of functional distractors/3) × 100. Accordingly, DE values were 100%, 66.6%, 33.3%, and 0% for 3, 2, 1, and 0 functional distractors, respectively [1]. Responses were coded as 1 for correct and 0 for incorrect and entered into Microsoft Excel 2013 (Microsoft Corp., USA) for coding and analysis. Descriptive statistics (mean, proportions) were used to summarize item indices and test performance. Items were categorized for retention, revision, and discard based on predefined desired criteria. Items with moderate difficulty, acceptable to good discrimination, and all three functional distractors were retained. Items outside these criteria, i.e., very easy/very difficult, poor discrimination, or items with one or more non-functional distractors, were recommended for revision. Items demonstrating negative discrimination (DI< 0) were categorized for discard, as such items are considered defective and unsuitable for inclusion in the question bank without a complete rewrite. All indices and cut-offs used in this study (categories of difficulty index, extreme-group method of DI, and distractor efficiency using non-functional distractors defined as <5% selection) are derived from published literature and do not require any license for use [1,6,7,8].

## Results

A total of 138 seventh-semester MBBS students completed the ENT part completion test (30 single best answer MCQs; maximum score was 30). The mean total score was 10.76 ± 5.36 with a range of 4-28. Item-level performance indices and item decisions are provided in Table 1. Overall, based on combined indices, 10 items were retained without modification, 17 were recommended for revision, and three items were discarded, as shown in Table 1.

**Table 1:** Item-wise analysis summary

Item No.	Difficulty index (p-value)	Discrimination index (DI)	Distractor efficiency (%)	Decision
Q1	0.283	0.892	66.67	Revise
Q2	0.246	0.703	100.00	Revise
Q3	0.225	0.757	100.00	Revise
Q4	0.275	0.730	100.00	Revise
Q5	0.442	0.486	100.00	Retain
Q6	0.514	-0.162	100.00	Discard
Q7	0.833	-0.081	66.67	Discard
Q8	0.681	0.297	100.00	Retain
Q9	0.072	0.135	100.00	Revise
Q10	0.246	0.730	100.00	Revise
Q11	0.659	0.324	100.00	Retain
Q12	0.362	0.757	100.00	Retain
Q13	0.645	0.270	100.00	Retain
Q14	0.225	0.486	100.00	Revise
Q15	0.304	0.811	100.00	Retain
Q16	0.587	0.378	100.00	Retain
Q17	0.174	0.514	66.67	Revise
Q18	0.130	0.351	100.00	Revise
Q19	0.232	0.541	100.00	Revise
Q20	0.210	0.757	100.00	Revise
Q21	0.275	0.730	33.33	Revise
Q22	0.449	0.432	100.00	Retain
Q23	0.449	0.405	100.00	Retain
Q24	0.377	0.459	66.67	Revise
Q25	0.580	-0.135	33.33	Discard
Q26	0.348	0.351	100.00	Retain
Q27	0.080	0.108	100.00	Revise
Q28	0.464	0.324	66.67	Revise
Q29	0.196	0.297	100.00	Revise
Q30	0.196	0.108	100.00	Revise

**Figure 1:**
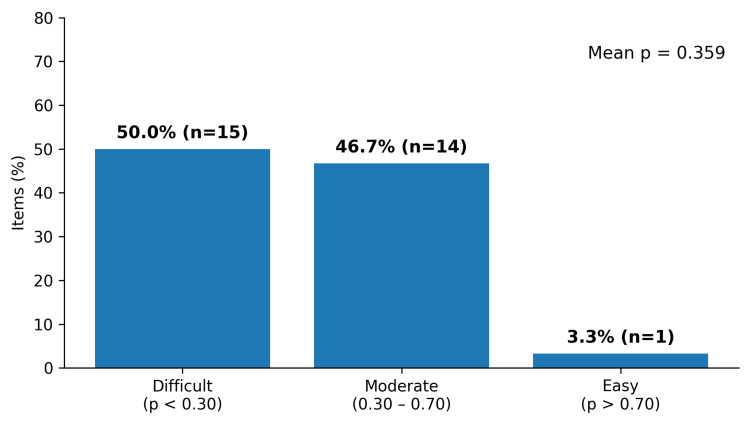
Distribution of item difficulty (p value) categories (n = 30) Reference: [6]

The mean difficulty index was 0.359 (range 0.072- 0.833), with a distribution skewed towards greater difficulty. Figure 1 shows that 15/30 items (50.0%) were difficult (p < 0.30), 14/30 items (46.7%) were moderate difficulty (0.30-0.70), and 1/30 item (3.3%) was easy.

**Figure 2:**
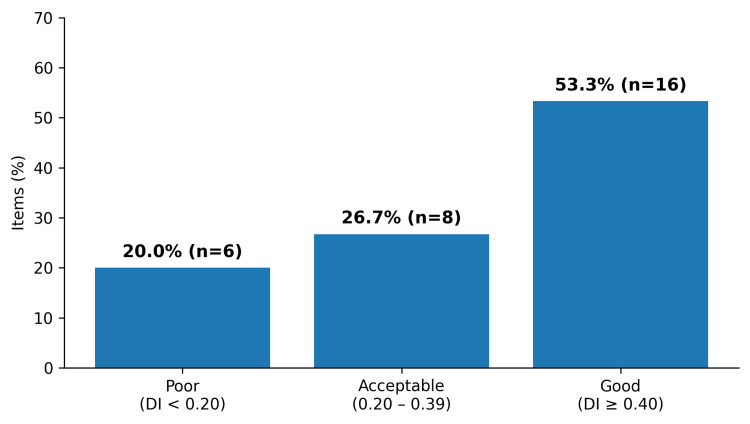
Distribution of discrimination index (DI) categories (n = 30) References: [2,7,8]

Discrimination performance is summarized in Figure 2. Sixteen items (53.3%) achieved good discrimination (DI ≥ 0.40), and eight items (26.7%) demonstrated acceptable discrimination (0.20-0.39). Six items (20.0%) showed poor discrimination (<0.20) between students, including three items (Q6, Q7, and Q25) that had negative discrimination. These three items having (DI < 0) were discarded as per prespecified decision rules.

**Figure 3:**
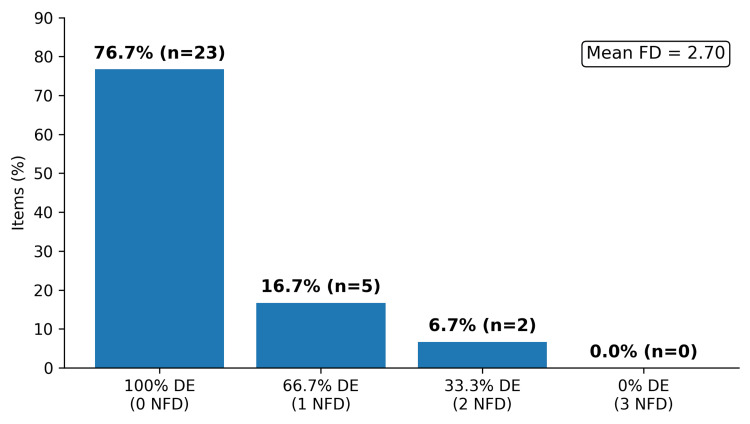
Distractor efficiency (DE) categories (n = 30) Reference: [1]

Distractor performance is summarized in Figure 3. Twenty-three items (76.7%) had three functional distractors (DE = 100%), five items (16.7%) had two functional distractors (DE = 66.7%), and two items (6.7%) had one functional distractor (DE = 33.3%). There was no item with all three non-functional distractors. The mean distractor efficiency was 90%.

**Figure 4:**
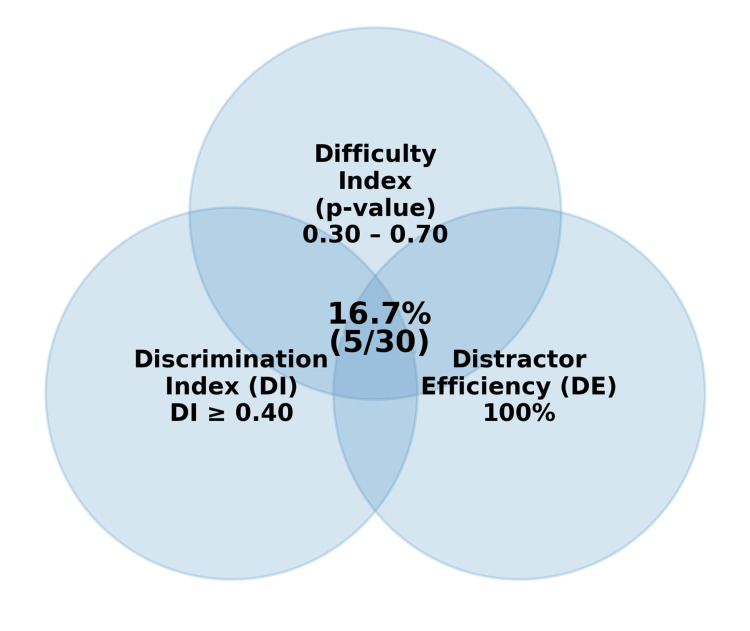
Venn diagram showing items meeting all three criteria (schematic) References: [1,2,6,7,8]

Overlap of items meeting all three desired criteria, i.e., moderate difficulty, good discrimination (DI ≥ 0.40), and DE = 100%, is shown in Figure 4. Five items (5/30; 16.7%) satisfied all three criteria.

## Discussion

This post-test item analysis of an ENT internal assessment of the MCQ paper demonstrated a relatively difficult test overall, with a mean difficulty index of 0.359 and 50% of items categorized as difficult. At the same time, more than half of the items showed good discrimination (DI ≥ 0.40) and strong distractor functioning (76.7% of items with three functional distractors). The 90% distractor efficiency of the present study is close to that reported by Chauhan et al. (84%) [6] and Patil et al. (82.8%) [2]. It is higher than that reported by Khilnani (ENT) et al. (74.6%) [9]. This was substantially higher than that reported by Tarrant et al., where only 13.8% items had three functional distractors [1]. It suggests that the distractors are plausible and attractive, but it also contributes to the overall greater difficulty level, which is a known interaction. The difficulty index of the present study is close to that reported by Patil et al. (0.383) [2] but lower than that reported by Namdeo and Sahoo (0.659) [4]. Similarly, Adiga et al. reported difficulty indices of 0.60, 0.75, and 0.90 in their three tests [5]. Together, these comparisons suggest that the test had a greater proportion of difficult items. The DI of the present study (mean DI = 0.425) compares favorably with Chauhan et al. (mean DI = 0.44) [6] and is higher than Namdeo and Sahoo (mean DI = 0.33) [4], Patil et al. (mean DI = 0.27) [2], and Gajjar et al. (mean DI = 0.14) [10]. This pattern indicates that although the paper was harder, many items still separated high and low achieving students effectively, which is a key indicator of item quality. The difficulty index, DI, and distractor efficiency should be interpreted together rather than in isolation, because difficult items may still show good discrimination, and high distractor efficiency may contribute to greater overall item difficulty. In the present study, only five items (5/30; 16.7%) met all three predefined desired criteria, indicating that acceptable performance on one index did not always correspond to optimal performance on the others. Formal analysis of the relationship among these indices was beyond the scope of the present study and may be considered in future work. The mean total score of 10.76 out of 30 suggests that the examination was relatively challenging for this cohort.

In the present study, the main purpose was item-level post-test analysis rather than pass-fail interpretation. The relatively low mean score may reflect the higher overall difficulty level of the test and supports the need for a structured review of item quality after the examination. The DI was calculated using the extreme-group method. Patil et al reported 3/30 MCQs meeting predefined desired criteria [2], whereas the present study found 5/30 items (16.7%) meeting all predefined desired criteria for MCQs. Items with negative DI should be carefully reviewed before any future reuse and should be completely revised. The three items with negative discrimination (Q6, Q7, and Q25) are particularly important because such items may fail to distinguish high-achieving students from low-achieving students. Negative discrimination may indicate problems such as ambiguous wording, miskeying, confusing distractors, or poor alignment with the taught content. Future papers should increase the proportion of MCQs with moderate difficulty while maintaining discrimination. High distractor efficiency should be maintained through systematic distractor review. The present study uses standard item indices (difficulty index, DI, and distractor efficiency), which give objective and actionable feedback for item bank improvement. The clear retain/ revise/ discard rules enable direct quality improvement decisions. A moderate cohort (n = 138) improves the stability of item indices compared with smaller samples. The present study is from a single institute, single subject, and a single test, so it has limited generalizability. The definition of non-functional distractors (selection < 5%) is frequency-based and may miss option-level discrimination issues. The analysis was restricted to post-test item statistics and did not include additional psychometric measures such as point-biserial correlation or item-total correlation. Internal consistency reliability measures, such as KR-20 or Cronbach's alpha, were not computed in the present study; therefore, the overall reliability of the test could not be quantified [11,12]. Educational measurement literature recommends reporting internal consistency alongside item indices to strengthen psychometric interpretation and item-bank decisions [13,14]. Future assessments should report reliability estimates to improve the reproducibility of assessment data [15].

## Conclusions

Many items in the present study showed good discrimination with strong distractor functioning, supporting their usefulness in assessment. However, the paper was skewed towards difficulty items, and three items showed negative discrimination. Future revision should increase the proportion of items with moderate difficulty while preserving good discrimination and functional distractors. Regular item analysis can strengthen the MCQ bank at the departmental level and support local assessment quality improvement.

## Appendices

MCQs Part Completion Test

1. Pinna develops from

a. 4st arch                        c. 1st and 2nd arch

b. 3rd arch                        d. 5th arch

2. Stapes suprastructure develops from

a. 1st arch                        c. 3rd arch

b. 2nd arch                        d. 4th arch

3. Santorini’s fissure is present in/ between

a. Cartilaginous canal                    c. Between the inner and middle ear              

b. Bony canal                                  d. Between the middle ear and pharynx

4. Ceruminous glands are 

a. Modifies accrine                          c. Modified apocrine

b. Holocrine                                      d. Modified holocrine

5. Thickness of tympanic membrane

a. 0.1 mm                                           c. 0.3 mm

b. 0.2 mm                                           d. 0.4 mm

6. The major portion of the tympanic membrane is supplied by

a. Greater auricular nerve                            c. Arnold’s nerve

b. Lesser occipital nerve                              d. Facial nerve

7. Jacobson’s nerve is a branch of

a. VII nerve                                           c. IX nerve

b. X nerve                                              d. Greater auricular nerve

8. Cough on scratching the EAC is due to

a. Auriculotemporal nerve                     c. Greater auricular nerve

b. X nerve                                                d. VII nerve

9. Referred ear pain due to pathology in the vallecula is by

a. X nerve                                                c. V nerve

b. IX nerve                                               d. VII nerve

10. The narrowest portion of the middle ear in the transverse section is

a. Epitympanum                                      c. Hypotympanum

b. Mesotympanum                                  d. Endotympanum

11. A dehiscent middle ear floor can lead to injury of

a. Internal carotid artery                            c. Parotid gland

b. Internal jugular vein                              d. Mastoid

12. Tegmen tympani is

a. Roof                                                       c. Medial wall

b. Floor                                                      d. Lateral wall

13. The type of joint present between the ossicles is

a. Primary cartilaginous                                 c. Synovial

b. Secondary cartilaginous                             d. Fibrous

14. The round window is covered by

a. Footplate of stapes                                     c. TM

b. Secondary TM                                           d. Reissner’s membrane

15. Commonest route of infection in AOM:

a. Hematogenous                                                c. Tympanic membrane

b. Eustachian tube                                              d. Cutaneous lymphatics

16. The most common organism in AOM:

a. Beta hemolytic Streptococci                            c. H. influenza

b. S. pneumonia                                                    d. S. aureus 

17. Most common site for perforation of the tympanic membrane in AOM:

a. Superior half of pars tensa                                       c. Posterior half of pars tensa

b. Anterior half of pars flaccida                                   d. Superior half of pars flaccida

18. Origin of cholesteatoma is not explained by  

a. Wittmaack’s theory                                                      c. Habermann’s theory

b. Ruedi’s theory                                                              d. von Bekesy theory

19. Primary acquired cholesteatoma occurs in the majority of cases in

a. Mastoid antrum                                                          c. Facial recess

b. Sinus tympani                                                             d. Prussack’s space

20. Surgical intervention was done for COM mucosal disease

a. Simple mastoidectomy                                       c. Modified radical mastoidectomy

b. Radical mastoidectomy                                      d. Tympanoplasty

21. Wullstein’s classification is used for

a. Tympanoplasty                                                             c. Ossiculoplasty

b. Myringoplasty                                                               d. Meatoplasty

22. Otosclerosis affects

a. Otic labyrinth                                                              c. Otic capsule

b. Peri-otic labyrinth                                                       d. Peri-otic capsule

23. What type of hearing defect is not seen in otosclerosis?

a. Paracusis willisii                                           c. Progressive conductive hearing loss

b. Presbycusis                                                   d. SNHL.

24. Which symptom is least experienced by a person with Meniere’s disease?

a. Episodic vertigo                                                    c. Fluctuating hearing loss

b. Otalgia                                                                   d. Tinnitus

25. In patients diagnosed with Meniere’s, intolerance to loud sounds is by

a. Recruitment phenomena                                       c. Diplacusis

b. Rollover phenomena                                              d. Tullio phenomena

26. During Valsalva, the structure aiding in the opening of the eustachian tube is

a. Tensor tympani                                                       c. Stylopharyngeus

b. Tenosr veli palatini                                                 d. Palatopharyngeus

27. Flat non-ciliated epithelium is present in

a. Eustachian tube                                                    c. Mesotympanum

b. Hypotympanum                                                   d. Mastoid air cells

28. Largest mastoid air cell

a. Attic                                                                              c. Antrum

b. Aditus                                                                           d. Atrium

29. The supra-metatarsal triangle is formed by all except

a. Temporal line                                                          c. Tangent drawn to EAC

b. Posterior segment of EAC                                       d. Mastoid process

30. Citelli’s angle is present between

a. Tympanic membrane and horizontal                          c. Styloid and vertical

b. 3rd and 4th part of facial nerve                                     d. Sigmoid sinus and tegmen.

## References

[1] Tarrant M, Ware J, Mohammed AM (2009). An assessment of functioning and non-functioning distractors in multiple-choice questions: a descriptive analysis. BMC Med Educ.

[2] Patil R, Palve SB, Vell K, Boratne AV (2016). Evaluation of multiple-choice questions by item analysis in a medical college at Pondicherry, India. Int J Community Med Public Health.

[3] Haladyna TM, Downing SM, Rodriguez MC (2002). A review of multiple-choice item-writing guidelines for classroom assessment. Appl Meas Educ.

[4] Namdeo SK, Sahoo S (2016). Item analysis of multiple-choice questions from an assessment of medical students in Bhubaneswar, India. Int J Res Med Sci.

[5] Adiga MNS, Acharya S, Holla R (2021). Item analysis of multiple-choice questions in pharmacology in an Indian medical school. J Health Allied Sci NU.

[6] Chauhan GR, Chauhan BR, Vaza JV, Chauhan PR (2023). Relations of the number of functioning distractors with the item difficulty index and the item discrimination power in the multiple- choice questions. Cureus.

[7] Kelley TL (1939). The selection of upper and lower groups for the validation of test items. J Educ Psychol.

[8] Cureton EE (1957). The upper and lower twenty-seven per cent rule. Psychometrika.

[9] Khilnani Khilnani, A. K., Thaddanee Thaddanee, R. R., & Khilnani, G. (2019 (2019). Development of multiple-choice question bank in otorhinolaryngology by item analysis: a cross-sectional study. Int J Otorhinolaryngol Head Neck Surg.

[10] Gajjar S, Sharma R, Kumar P, Rana M (2014). Item and test analysis to identify quality multiple choice questions (MCQs) from an assessment of medical students of Ahmedabad, Gujarat. Indian J Community Med.

[11] Cronbach LJ (1951). Coefficient alpha and the internal structure of tests. Psychometrika.

[12] Kuder GF, Richardson MW (1937). The theory of the estimation of test reliability. Psychometrika.

[13] Tavakol M, Dennick R (2011). Making sense of Cronbach's alpha. Int J Med Educ.

[14] Ebel RL, Frisbie DA (1991). Essentials of Educational Measurement. Prentice Hall.

[15] Downing SM (2004). Reliability: on the reproducibility of assessment data. Med Educ.

